# Engineering a dually perfused miniaturized bioreactor platform to enable multi-tissue knee joint modeling in vitro

**DOI:** 10.1016/j.jcjp.2026.100328

**Published:** 2026-10-17

**Authors:** Núria Ginés Rodriguez, P.N. Bernal, E. Miralles Garcia, J. Malda, R. Levato

**Affiliations:** a Department of Orthopaedics, University Medical Center Utrecht, Utrecht University, Utrecht, The Netherlands; b Regenerative Medicine Center Utrecht, Utrecht, The Netherlands; c Department of Clinical Sciences, Faculty of Veterinary Medicine, Utrecht University, Utrecht, The Netherlands

**Keywords:** Articulating joints, Bioreactors, Disease modeling, Microfluidics, Osteoarthritis

## Abstract

**Introduction:**

Osteoarthritis (OA) is a leading cause of disability worldwide, with incidence rates increasing due to ageing populations. Despite its growing societal and health care burden, no disease-modifying therapies are currently available, and treatment remains limited to symptom management or surgical intervention in advanced disease. Human-relevant in vitro models are therefore critically needed to study early disease onset and predict therapeutic outcomes.

**Objectives:**

This study aimed to develop a dually perfused in vitro bioreactor platform incorporating 4 relevant knee joint compartments to support early-onset OA disease modeling and drug testing.

**Methods:**

Triply periodic minimal surface trabecular bone structures were engineered using volumetric bioprinting. A biomaterial-based strategy was evaluated to mimic the transport function of the osteochondral interface. Synovial fibroblast culture conditions were optimized, while articular cartilage formation from articular chondroprogenitor cells was assessed under synovial cell culture conditions. Finally, the compartments were assembled into a dual-perfusion bioreactor and evaluated for feasibility of integrated culture and perfusion.

**Results:**

Volumetric bioprinting enabled the fabrication of complex, mineralized trabecular bone-like structures. The biomaterial interface supported size-dependent, inter-compartmental transport, while a lubricin- and Cadherin-11-expressing synovial fibroblast layer was achieved. Articular chondroprogenitors supported cartilage-like tissue formation under synovium-compatible culture conditions. Perfusion studies demonstrated the feasibility of integrating the 4 compartments within a single bioreactor platform.

**Conclusions:**

This work establishes a technological foundation for a multicompartment joint in vitro model incorporating key features of bone, osteochondral interface, synovium, and cartilage. With further biological validation, this platform holds promise for modeling early OA and evaluating human-relevant therapeutic responses.

## Introduction

Osteoarthritis (OA) is the most common musculoskeletal disease and a leading cause of disability worldwide. It affects more than 500 million people globally, with a 9.3% increase in incidence between 1990 and 2017. In 2020, the global incidence of knee OA was estimated at 86.7 million people.^1–3^ OA prevalence is substantially higher in women than in men and has increased by 132.3% over the past 30 years. Additional established risk factors for OA include high BMI, sedentary lifestyle and prior traumatic injury to the joint capsule.^4^ OA is a highly prevalent and costly disease for affected individuals and health care systems, making its study and the development of appropriate in vitro models of paramount importance. Many existing models have focused primarily on the cartilaginous component of the disease.^3^ However, OA affects the entire joint, involving pathological changes in the subchondral bone, synovial inflammation and damage to other joint structures.^2,5,6^ A wide range of in vitro models have been described, spanning from 2D cultures of primary cells to engineered 3D constructs.^7–9^

More recently, microfluidic models of the knee joint and OA have gained attention as they enable the recapitulation of a dynamic and controllable tissue microenvironment that can be tuned to reflect native joint properties.^10–15^ Several studies have combined cartilage explants or differentiated primary chondrocytes with synovial cells in microfluidic platforms to model aspects of the knee joint.^16^ Notably, Lin et al developed a dually perfused induced pluripotent stem cell (iPSC)-derived model in which the formation of an osteochondral interface was observed under dynamic conditions.^13^ Zhong et al described a microfluidic model of the knee joint comprised of engineered bone-cartilage, synovium and adipose tissue in a joint-on-chip array.^15^ These studies further show the implementation of knee-joint and OA in vitro models toward the use of advanced fabrication techniques and interconnection of 2 or more tissues present in the knee joint while proving the feasibility to maintain lengthy differentiation protocols in dynamic conditions.^12,17–19^ Despite these advances, few reports describe dynamic models incorporating more than 2 joint tissues, restricting the study of multi-tissue crosstalk underlying joint homeostasis and early OA onset. Herein, we report on the fabrication of a dually perfusable bioreactor platform that incorporates trabecular bone, an osteochondral interface, articular cartilage and a synovium compartment.

## Materials and methods

### Cell culture

Human bone marrow-derived mesenchymal stromal cells (hMSCs) were isolated from bone marrow aspirates of consenting patients, as previously described.^20^ Human bone marrow aspirates were obtained from the iliac crest of patients who were receiving spondylodesis or hip replacement surgery. Isolation and distribution were performed in accordance with protocols approved by the Biobank Research Ethics Committee (isolation 08-001, distribution protocol 18-739, University Medical Centre Utrecht). The protocols used were in line with the principles embodied in the Declaration of Helsinki. MSCs were expanded in α-Modified Eagle Medium culture medium (α-MEM, Gibco) supplemented with 10% fetal bovine serum (FBS; Capricorn Scientific), 1% Penicillin/Streptomycin (P/S; ThermoFisher Scientific), 1% L-ascorbic acid-2-phosphate (ASAP; Sigma-Aldrich), and 1 ng/mL^−1^ basic fibroblast growth factor (bFGF; R&D Systems) and used at passage 4 to 5.

Articular cartilage progenitor cells (ACPC): Osteoarthritic cartilage was obtained from redundant material from patients undergoing total knee arthroplasty. Anonymous collection of this tissue was performed according to medical ethics regulations of the University Medical Center Utrecht and the guideline “good use of redundant tissue for research” of the Dutch Federation of Medical Research Societies.^21^

Progenitor cells were isolated via a fibronectin adhesion assay as previously described[ref]. Briefly, freshly isolated cartilage cells were plated in fibronectin-coated tissue culture plates, at a density of 500 cells cm^−2^. After 20 minutes, non-adherent cells were removed, and the attached cells were cultured in Dulbecco’s Modified Eagle Medium (DMEM), 10% FBS, 1% P/S, 200 μM ASAP, and 5 ng/mL bFGF.^21,22^ ACPC differentiation toward chondrogenic lineage was performed using DMEM, supplemented with 1% ASAP, 1% P/S, 1% ITS+ premix (Corning), 1% HEPES (ThermoFisher Scientific), 0.004% dexamethasone (Sigma-Aldrich), and 1 μL/mL Transforming Growth Factor (TGF-β1, Peprotech).

Synovial fibroblast-like cells were isolated from synovial tissues obtained as discarded specimen from (OA) patients undergoing total joint arthroplasty at the University Medical Centre Utrecht and according to the institutional Medical Ethical regulations as previously described and cultured in DMEM/F-12 medium, supplemented with 10% FBS, 1% P/S, and 1% ASAP. Cells were used at passage 4 to 6.^21,23,24^

### Bone mimetic model preparation

BM-hMSC were volumetrically bioprinted (VBP, Tomolite 1, Readily 3D) into a Schwarz G triply periodic minimal surface (TPMS) structure of 10 mm length and 6.6 mm diameter. Prints were performed with 5 × 10^6^ hMSCs/mL embedded within 5% w/v gelatin methacryloyl (GelMA; 93% degree of functionalization) supplemented with 0.1% w/v lithium phenyl(2,4,6-trimethylbenzoyl) phosphinate (LAP, Tokyo Chemical Industry) as photoinitiator and 10% v/v iodixanol (Optiprep, Cell StemCell) to mitigate light scattering during printing (light dose = 150 mJ/cm^2^, printing time = 15 seconds).^25^ Prints were post-cured in a UV-oven (CL-1000, Ultraviolet Crosslinker, λ = 365 nm, I = 8 mW/cm^2^; UVP,) for 5 minutes. The volumetrically printed TPMS structures were differentiated toward osteogenic lineage using Osteogenic differentiation medium bulletKit (Lonza). Day 1 samples were imaged label-free in a custom-made lightsheet microscope. Day 1 and 21 differentiated samples were evaluated for calcification via micro-computed tomography (microCT, CT lab GX, Rigaku, using software from PerkinElmer) at a voltage of 90 kV, current 180 mA and FOV = 15.

### Glycosaminoglycan (GAG) quantification

Chondrogenic differentiation samples were freeze-dried and digested overnight in 0.01 M cysteine (Sigma-Aldrich), 250 μg/mL papain (Sigma-Aldrich), 0.2 M NaH_2_ PO_4_ and 0.01 M EDTA at 60 °C. Digests were then reacted with dimethylmethylene blue (DMMB, Sigma-Aldrich), and the sGAG content calculated by determining the ratio of the absorbance at 525 and 595 nm with a CLARIOstar Plus spectrophotometer (BMG Labtech). Known dilutions of chondroitin sulfate were used as standards for quantitative analysis. sGAG concentrations were normalized against double stranded DNA concentration, obtained using a Quant-iT PicoGreen dsDNA kit (Life Technologies), used according to manufacturer’s protocol.

### Histology

Samples were fixed in formalin and embedded in paraffin prior to sectioning (5 μm sections). Light microscopy images of stained samples were acquired using a BX51 microscope (Olympus). Mineralization of osteogenically differentiated samples was assessed using Alizarin Red staining on paraffin sections (2% Alizarin Red for 2 minutes). Proteoglycan and collagen deposition of chondrogenically differentiated samples was assessed though Safranin-O and Fast green staining (0.125% Safranin-O for 5 minutes; 0.4% Fast Green for 4 minutes).

Collagen type I and II in chondrogenic samples were also visualized via immunohistochemistry (IHC). As previously described in our lab using primary antibodies (rabbit anti-collagen type I, 1:400, Abcam, ab138492; mouse anti-collagen type II, 1:100, DHSB, II-II6B3) and secondaries, collagen type I, the BrightVision goat anti-rabbit horseradish peroxidase (HRP)-conjugated antibody (Nordic Immunologic Laboratories) and for collagen type II, a rabbit anti-mouse immunoglobulins/HRP-conjugated antibody (1:100, Agilent Technologies).^26^

### Immunofluorescence

Synovial samples were stained for key tissue markers. Samples were fixed permeabilized in 0.2% Triton X-100 in PBS and blocked in 5% BSA in PBS. Primary antibodies were diluted in 5% BSA (1:250 Cadherin 11 (CDH11 71-7600 Rabbit a-human, Thermo Fisher), 1:250 PRG4 (anti-lubricin, clone 9G3 mouse a-human, Merck)). Antibodies were added to the respective samples and incubated at 4 °C overnight. Secondary antibodies were diluted in 5% BSA (1:150 goat anti-rabbit A488, 1:150 goat anti-mouse A640). Samples were incubated for 3 hours at RT and imaged using a Thunder microscope (Leica).^27^

### Transport through the engineered osteochondral interface

Iodixanol transport: Disks of 20% w/v GelMA and 20% w/v poly(ethylene glycol) diacrylate (PEGDA; M_n_700, Sigma-Aldrich) were casted in 6 × 2 mm teflon molds using 0.1% LAP as photoinitiator. Disks were imaged in a microCT scanner (voltage = 90 kV, current = 180 mA, FOV = 15). A droplet of iodixanol (MW = 1550 g/mol) was added on top of the preformed hydrogels, and samples were imaged after 0, 15, 30, 60, 90, and 120 minutes. To quantify iodixanol diffusion through the crosslinked hydrogels, the scan intensity was quantified via Fiji (Image J) on cross-sectional images of the hydrogels after background subtraction. Measurements were taken at the 1 mm midpoint and represented as a % of initial (or maximum) gray intensity.

FITC-Inulin: Disks of 20% w/v GelMA and 20% w/v PEGDA were cast on the membrane side of 9 mm diameter Transwell inserts (Merck) and placed in a 12-well plate. Hydrogel-free Transwell were used as negative controls. The lower side of the well was filled with 2 mL PBS while the upper Transwell compartment was filled with a solution of FITC-Inulin (500 μg/mL in PBS, MW = 2 g/mol, TbD Labs). The plate was incubated at room temperature and in the dark for 120 minutes. 50 μL samples were taken from the lower side every 15 minutes for 120 minutes to quantify FITC-Inulin fluorescence (490/520 nm; ClarioStar) and normalized by a blank of PBS.

### Fabrication of the bioreactor

All bioreactor parts were custom designed in Fusion360 (AutoDesk). The inner compartment holding the hydrogels was fabricated by producing a negative mold in a FormLabs 3B+ SLA printer using Gray resin (FormLabs). Polydimethylsiloxane (PDMS, SYLGARD 184, Dow Corning) was poured into the mold and cured for 2 hours at 80 °C. The 2-part outer casing of the bioreactor was printed in an FDM BambuLabs XC-1 Carbon (BambuLabs) using polyethyleentereftalaatglycol (PETG). M4 threaded nuts and matching M4 bolts were used to screw the PETG casing shut and seal the bioreactor chamber, encasing the PDMS between 2 glass-slides for optimal optical access.

### Microbead perfusion setup

The platform was assembled, incorporating cell-free hydrogel components to assess compartmentalized perfusability. The inlets and outlets were connected to a peristaltic pump (ISMATEC Regio ICC, 12 rollers, 3-stop tubing, ID 2.54 mm) using G17 needles (Nordson) and PTFE tubing (1/16” outer diameter; Darwin Microfluidics). Microbeads (fluorescent green polyethylene microspheres, 125-150 μm and 450-500 μm in diameter; Cospheric) were suspended in 0.1% Tween 20 in PBS and perfused through the bone compartment at 150 μL/h (125-150 μm) and through the cartilage/synovium compartment at 400 μL/h (450-500 μm). The microbeads were illuminated with 405 nm LED lamp and imaged with a Basler ace2 a2A1920-160umBAS Monochrome USB3 Basic (Basler, Edmund Optics) camera with Lens C125-2522-5M-P f25 mm lens with a bandpass filter 530 ± 40 nm central wavelength and corresponding software.

### Statistical analysis

The normality of the data was quantified via the Shapiro-Wilk test. Statistical significance was quantified via 1 or 2-way ANOVA, along with multiple comparisons, were conducted using GraphPad Prism. Results were reported as mean ± standard deviation; statistical significance is indicated by *P <.05, **P <.01, ***P <.001, ****P <.0001. The corresponding figure legends indicate the sample size (*n*).

## Results and discussion

### Volumetric bioprinting (VBP) allows the fabrication of macroporous, cell-laden hydrogel networks that support osteogenic differentiation

**Fig. 1:**
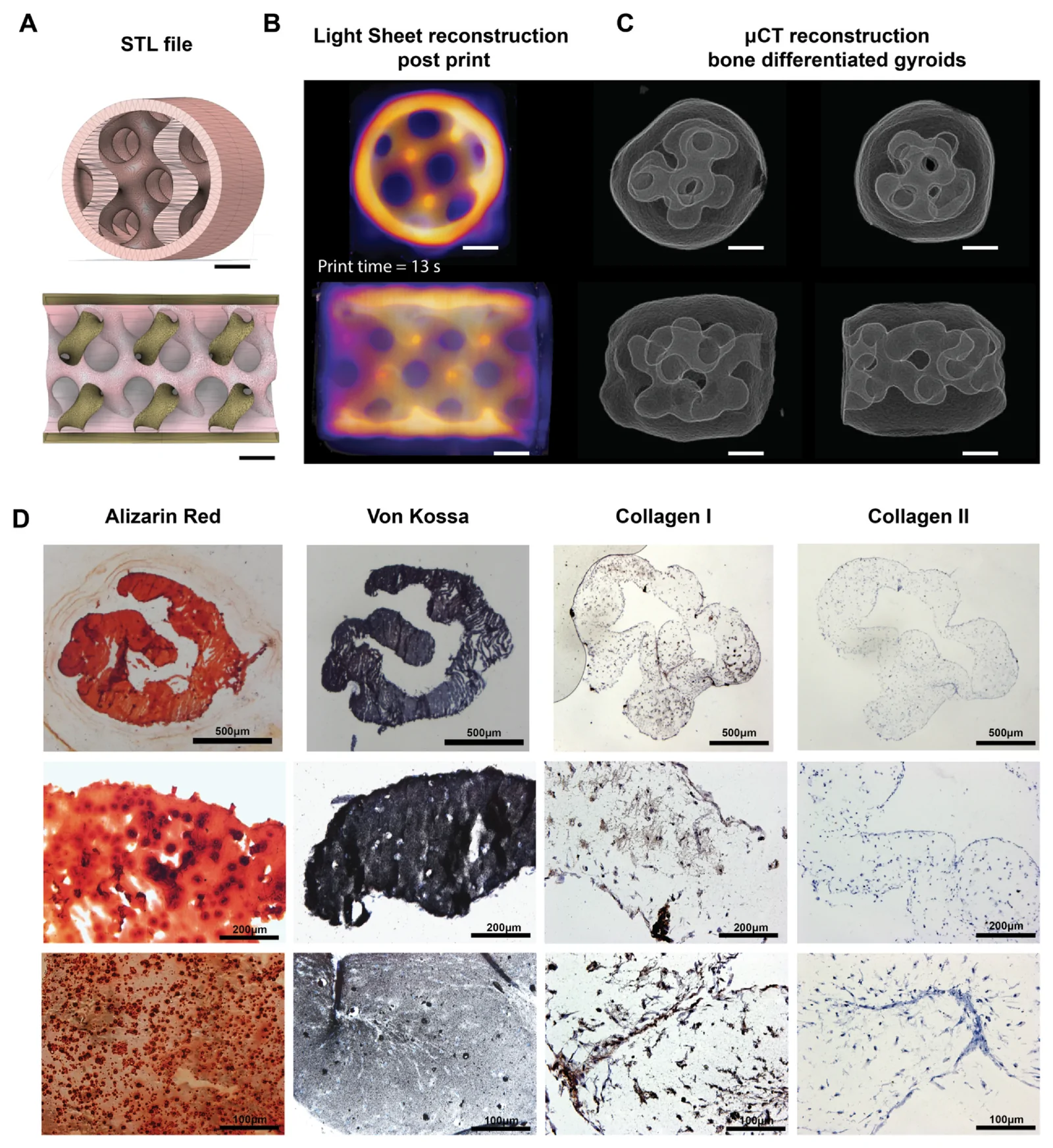
Osteogenic differentiation of volumetrically bioprinted (VBP) Schwarz G constructs. **A**, STL file of Schwarz G model for VBP. **B**, Lightsheet scan 3D render of cell-free construct immediately post-printing. *n* = 4. **C**, Day 21 microCT reconstruction of osteogenically differentiated human mesenchymal stromal cell (hMSC)-laden prints. *n* = 4. **A-C**, scalebar = 1 mm. **D**, Light microscopy images of VBP construct sections stained with Alizarin Red and Von Kossa to visualize calcium deposits and Collagen type I and type II after 21 d of osteogenic differentiation. *n* = 4.

VBP enables the rapid fabrication of centimeter-scale objects displaying high design freedom. Previous studies have demonstrated that distinct TPMS structures produced via VBP can modulate fluid flow and fluid dynamics through the hydrogel network, influencing cell behavior and functionality.^25^ Informed by previously published research,^28^ TPMS Schwarz-G-based structures (STL file, Fig. 1A) were printed via VBP and scanned via lightsheet microscopy (Fig. 1B), which showed the printed structures exhibited a complex and interconnected channel network. MSC-laden constructs were then osteogenically differentiated for 21 days and scanned via microCT to assess shape retention and still showed an interconnected, open pore network (Fig. 1C). Compared to non-differentiated controls, the differentiated samples macroscopically appeared white and opaque, and sufficiently stiff to withstand their own weight, indicating extracellular matrix deposition and mineralization had occurred throughout the culture period. Histological analysis confirmed the presence of calcification in the differentiated prints which showed positive Alizarin Red and Von Kossa staining all throughout the struts of the Schwarz G structure (Fig. 1D). The use of a macroporous structure resulted in homogeneous neo-matrix deposition and likely contributed to preventing the formation of a necrotic core in these centimeter-scale bioprinted constructs, in line with previous studies demonstrating the benefits of increased surface-area-to-volume ratios associated with such structures.^25,29,30^ The composition of the deposited extracellular matrix was revealed to contain collagen type I, while lacking collagen type II (Fig. 1D), indicative of the formation of a bone-like matrix. These results demonstrate successful osteogenic differentiation of cm-scale VBP constructs.^31^

### Engineered interface acts as a transport barrier between trabecular bone and articular cartilage

The osteochondral interface or calcified cartilage is a transitional layer between the deep zone of the articular cartilage and the subchondral bone. This layer is characterized by a transition of collagen type II to type I, a very low cell density and the lack of vascularization.^32,33^ To separate the bone and cartilage hydrogel compartments of the bioreactor, we aimed to build a cell-free structure which mimicked the transport properties of the native osteochondral interface. Nano-pores of 10.71 ± 6.45 nm in diameter have been identified in calcified cartilage with a disperse connectivity when compared to the subchondral plate with a limitation in the diffusion molecules above 2 kDa.^34^ PEGDA is a well-known hydrogel available in a range of molecular weights and tunable pore sizes depending on crosslinking density, reaching mesh sizes of around ~2 nm at a 20% w/v concentration.^35–37^ Here, PEGDA was compared against 20% GelMA (mesh size = 12-15 nm) as an interface material for regulating molecular transport. Two model probes were selected to probe size-dependent transport across the interface: iodixanol (MW 1.5 kDa), expected to gradually diffuse through the hydrogel layer, and FITC-Inulin (MW 2-5 kDa), expected to be largely retained on the probe-facing surface.

**Fig. 2:**
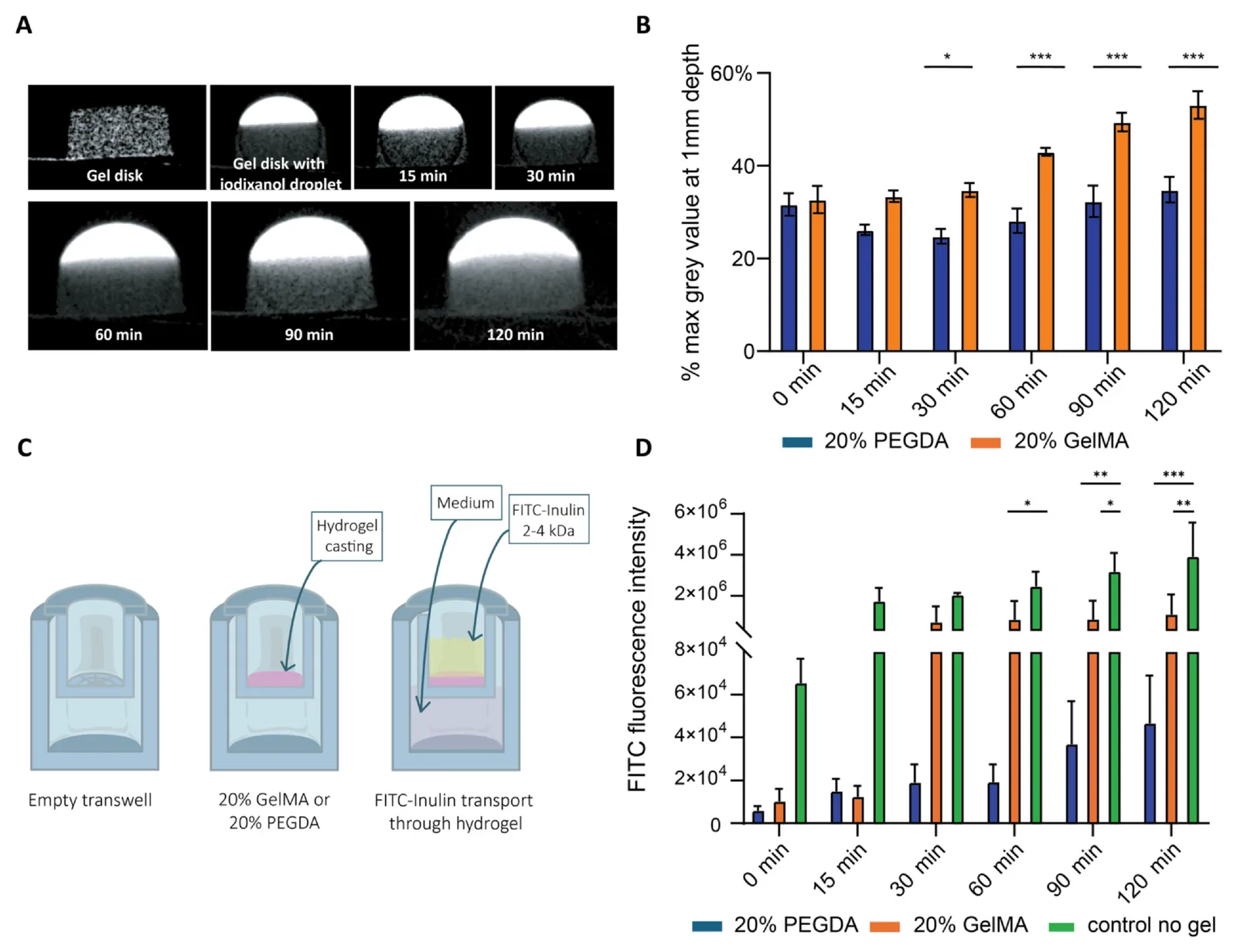
Transport assays to determine a molecular cutoff in the osteochondral interface. **A**, Iodixanol transport though hydrogel´s height at diverse timepoints. **B**, % of maximum gray value quantification at hydrogel depth of 1 mm, center point of the hydrogel, indicating the diffusion rate of iodixanol through the hydrogel over time, *n* = 4. **C**, Schematic of the FITC-Inulin hydrogel transport setup in Transwell. **D**, Quantification of the transport of FITC-Inulin through 20% polyethylene glycol diacrylate (PEGDA), 20% gelatin methacryloyl (GelMA) and controlled by an empty Transwell condition, *n* = 3.

To visualize and validate the transport behavior of small solutes across the hydrogel interface, iodixanol, previously used as a diffusion probe in hydrogel systems, was applied onto hydrogel disks, and its passive diffusion was monitored over time via microCT imaging (Fig. 2A).^34^ The results show the mean gray value at the midpoint of the hydrogel (Fig. 2B, 1 mm, % of maximum gray value across hydrogel width is shown in Supplementary Fig. 1) representing the rate at which iodixanol diffuses through the hydrogel. The faster the diffusion through the hydrogel, the more iodixanol would reach the center point of the hydrogel, therefore the higher the mean gray value at midpoint. At all timepoints of analysis, both hydrogel compositions were shown to be permissive to the passive transport of this low molecular weight probe, with PEGDA showing a slower transport rate than GelMA. The diffusion rate of PEGDA was slower than the diffusion rate of GelMA, likely due to PEGDA’s smaller mesh size. Both conditions showed a similar diffusion rate during the first 15 minutes of analysis with values reaching statistical significance at 30 minutes. From 30 minutes onward the amount of detected iodixanol at the midpoint of the hydrogel was significantly higher for GelMA compared to the PEGDA hydrogel composition. Overall, both tested hydrogel conditions were permissive to the passage of the 1.5 kDa probe but exhibited different diffusion rates correspondent to their nanoporosity.

The diffusion of molecules larger than the 2 kDa was also tested on the 2 materials using FITC-Inulin (2-5 kDa) as a fluorescent probe. Solute transport across the hydrogel was assessed using a Transwell-based diffusion assay (Fig. 2C). The progressive transport of the fluorescent compound through the hydrogel was quantified over time (Fig. 2D). Collected fluorescent data for timepoints 0 and 15 minutes showed a similar value between the GelMA and PEGDA conditions with a significant increase of the collected fluorescence on the GelMA samples at 60 minutes. The fluorescence detected for the GelMA samples peaked at minute 30 and then remained stable for the duration of the assay. This indicated that the transport of the fluorescent probe reached an equilibrium, as all FITC-Inulin permeated through 20% GelMA in 30 to 60 minutes. The diffusion of FITC-Inulin through PEGDA increased gradually throughout all assayed timepoints remaining 2 orders of magnitude lower and significantly different than 20% GelMA and the empty Transwell control. These results indicated that GelMA is highly permissive to the transport of molecules in the size range of 2 to 5 kDa and would not be suitable to mimic the selective diffusion observed in the native knee joint. Conversely, PEGDA showed significantly lower transport of FITC-Inulin through the hydrogel at 120 minutes incubation time, indicating a narrower diffusion range.

Since both tested hydrogel conditions were permissive to the passage of 1.5 kDa probe, but only 20% PEGDA restricted the diffusion of 2 to 5 kDa probes, the latter hydrogel was selected as a suitable component of the interface compartment between trabecular bone and engineered cartilage in the bioreactor platform.

### Cartilage maintains differentiation hallmarks in synovial medium

**Fig. 3:**
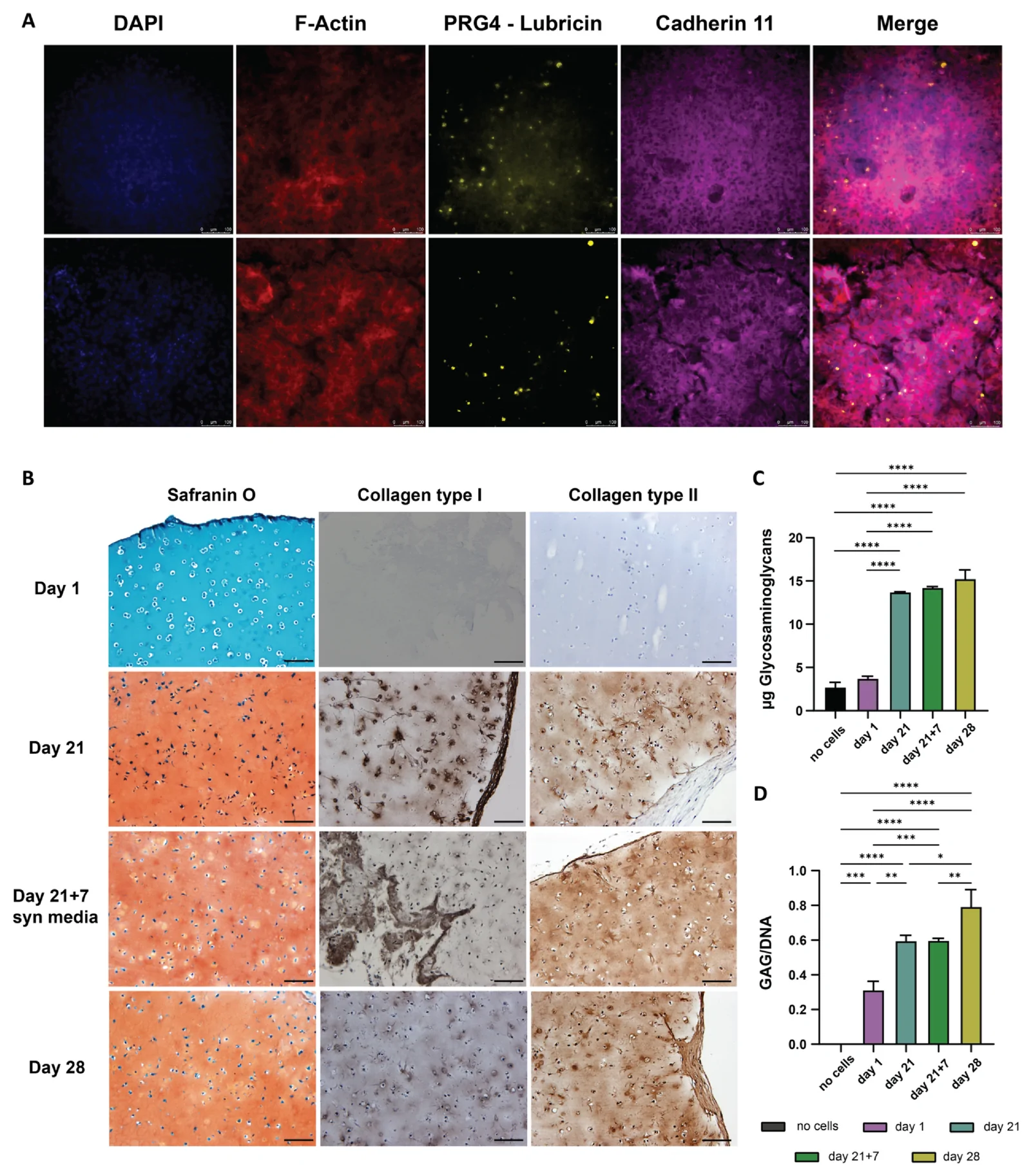
Culture and maintenance of the synovial and cartilage compartments. **A**, Representative fluorescence images of synovial-like fibroblasts cultured on the surface of 5% GelMA disks for a period of 10 d and imaged for Cadherin-11 and lubricin as a functional activity marker. Scale bar = 100 μm. Images taken with a Thunder microscope (Leica microsystems, *n* = 4). **B**, ACPC were cultured in chondrogenic medium for 21 d upon which they were either switched to synovial fibroblast medium (21+7) or kept in chondrogenic medium for another 7 d (28). Samples were then fixed and processed for histology, Safranin-O/FastGreen, immunohistochemistry of Collagen type 1 and Collagen type 2. Images taken in an Olympus BX51 microscope. Scalebar = 100 μm. Samples described in were processed for biochemical analysis (*n* = 3). **C**, Glycosaminoglycan (GAG) content *n* = 6, and (**D**) GAG/DNA *n* = 6.

The joint space is encased by the synovial capsule, there, a thin layer of synovial lining contains both fibroblasts and synovial macrophages that produce the synovial fluid.^38^ Synovial fluid is rich in hyaluronic acid, lubricin and aggrecan molecules which lubricate the cartilage layer and enable frictionless movement.^38,39^ As the synovial lining is on intimate but indirect contact with articular cartilage, it is of paramount importance to ensure chondrogenic differentiation is supported in the presence of synovial fibroblast medium. Synovial-like fibroblasts were cultured on 5% GelMA cast disks, their presence and functionality were tested via immunofluorescent staining for Cadherin-11, a cell-cell contact marker and for PRG-4/lubricin (Fig. 3A). After 10 days in culture, the cells formed a tight monolayer in the hydrogel’s surface which was seen in the staining for Cadherin-11 and F-actin, the cultured cells also produced lubricin which is a key function of synovial tissue.^38^

The ability of ACPC to generate articular cartilage-like tissue in GelMA-based hydrogels has previously been documented.^21,22,40^ Here, 21-day pre-differentiated cartilage disks (day 21) were cultured in either synovial fibroblast medium (day 21+7 samples) or chondrogenic medium for another 7 days (day 28 samples), all samples were stained for GAG deposition, collagen types I and II (Fig. 3B) and quantified for GAG production (Fig. 3C and D). Disks showed an increase in all positive staining from day 1 to day 21 which was maintained in further tested conditions. The results shown in the histological analysis correlate with biochemical assays for GAG quantification, which showed a significant increase over the culture time. Differentiated disks showed an increase in GAG/DNA content and displayed positive staining for Safranin-O and strong signal for collagen type 2 in contrast to non-differentiated controls. There was no significant difference in GAG deposition between differentiated conditions receiving synovial fibroblast medium or not. Nevertheless, there were significant differences in the GAG/DNA content for synovial medium and 28-day differentiated samples as well as 21-day differentiated samples, these differences can be explained by a decrease in DNA content by day 28 (Supplementary Fig. 2). Therefore, culturing pre-differentiated cartilage disks in synovial fibroblast medium proved to maintain differentiative hallmarks achieved during culture and resulted in similar expression of cartilage differentiative markers as samples cultured only in chondrogenic differentiation medium. These evidence supports the feasibility to culture both joint tissues within the same bioreactor compartment.

### Bioreactor assembly and microbead perfusion

**Fig. 4:**
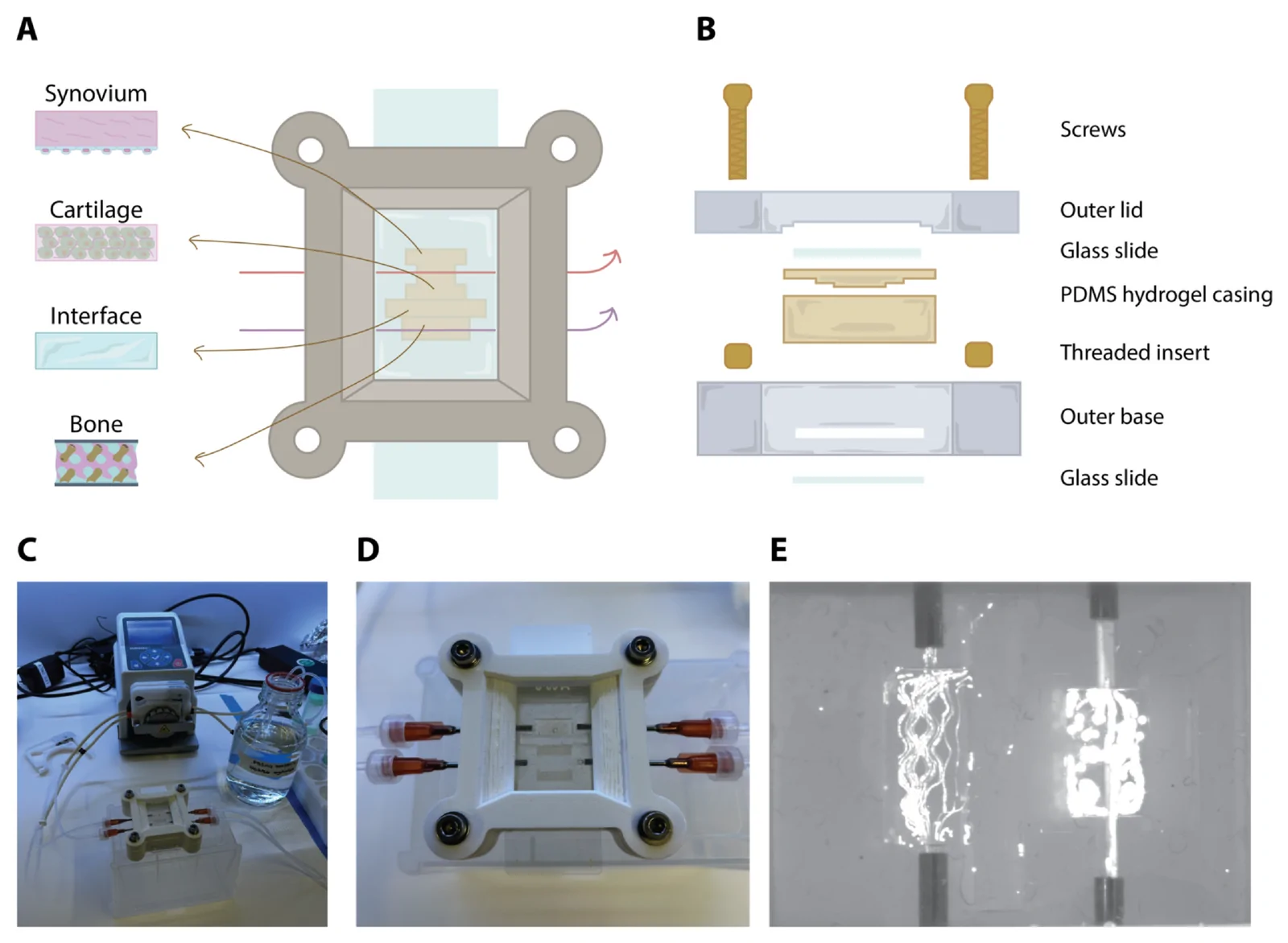
Assembly of the bioreactor platform. **A**, Schematic of top assembled view of the platform including the tissues intended in each slot of the inner holder. **B**, Expanded view of the components needed to assemble a sealed platform. **C**, Image of the perfusion setup. **D**, Image of the assembled platform containing all components, perfused with PBS. **E**, Max projection of 250 images of the perfusing microbeads, 125-150 μm through the VBP gyroid at 150 μL/h and 450-500 μm microbeads at 400 μL/h. Stacks analyzed via Fiji, Image J.

The hydrogel compartments were locally crosslinked in the inner PDMS chamber (5% GelMA for synovium and cartilage, 20% PEGDA for interface, and VBP bone construct inserted in the bone compartment). All compartments were cell-free, with the goal to test the ability of the bioreactor to permit leak-free independent perfusion of the osteal and synovial compartments (Fig. 4A). The platform with its hydrogel components was then assembled as shown in Figure 4B and connected to a peristaltic pump (Fig. 4C) after which it was flushed with PBS (Fig. 4D) prior to perfusion with fluorescent microbeads. Microbeads were perfused for 25 minutes, and image sequences were taken to reconstruct microbead paths (Fig. 4E). The platform could be assembled reproducibly, without presence of air bubbles and remained leak-free for a period of 48 hours at 37 °C. Figure 4E shows the passage of small microbeads through the microporous network of the Schwarz G structure shown in the lightsheet reconstruction of Figure 1B, proving that the VBP constructs presented open pores, were perfusable and could also osteogenically differentiate. Furthermore, the perfusion of the small microbeads appeared stable without any beads leaking from the system to the glass-slide compartment nor to the outer FDM-printed bioreactor casing.

The cartilage-synovium compartment was perfused with large microbeads that also showed continuous perfusion of the compartment without leaks or presence of the microbeads elsewhere outside the compartment. We also proved that pre-differentiated engineered articular cartilage could be cultured in synovial fibroblast medium, highlighting the potential integration success of those tissues in this described bioreactor platform.

## General discussion

Here, we have demonstrated the osteogenic differentiation of VBP MSC-laden gyroids, we have also proposed a material approach to the diffusion cut-off of the osteochondral interface in the knee joint, studied the culture of pre-differentiated engineered articular cartilage in synovial fibroblast medium and have provided a proof of concept of a dually perfusable bioreactor platform. The proposed bioreactor platform contains 2 independently perfusable lines and compartments that can contain highly cellularized hydrogel compositions and holds high promise to combine the 4 compartments, each mimicking key features of tissues present in the knee joint for modeling. Several studies have explored the combination of diverse tissues in the articular joint with special attention to the replication of the osteochondral unit. Other studies successfully integrated vascularised tissues in osteochondral systems to study the homing of immune cells through systemic perfusion and vascular hypertrophy during synovitis.^41–44^ These studies resulted in platforms containing physiologically relevant osteochondral units as vascularised trabecular bone with osteoclasts and osteoblasts as well as synovial capsule models to study the crosstalk between cartilage and synovial tissue and its response to several clinically relevant anti-inflammatory drugs.^42–44^ Such studies have shown that including different tissues and replicating the effects of external factors under dynamic conditions increases the predictability of OA in vitro models. A singular study has shown the integration of the osteochondral unit, synovium and fat pad and shown clinical relevance by validating both commercially available and experimental pharmacologic compounds.^15^

This project opens avenues in complex pharmaceutical modeling of whole joint diseases in a potentially personalized manner.

Nevertheless, the establishment of such a bioreactor platform comes with several challenges, including sensitivity to contamination and a lengthy assembly process, requiring multiple parts and steps. The platform is simultaneously perfused in 2 different channels, therefore requiring 2 different pump lines per platform and an extensive host of peripherals which limits the potential throughput of such a model. The system is internally separated by a biomaterial layer, upon long-term cell culture cells could infiltrate this divisor material and modify its mechanical properties, therefore hindering the optimized transport between bone and synovial compartments. Long-term differentiation culture under flow conditions has also been reported to be troublesome, the platforms become susceptible to air entrapment, bubble formation and movement of the peripherals, all increasing the risk of infection, movement, swelling or shrinking of the hydrogel components and loosening of the compartments.^41–43^ On the other hand, engineered modular systems hold promise to incorporate diverse cell sources as primary, patient derived and iPSC which would aid to develop personalized models of disease and screening for patient-dependent drug responses.^16,44–46^ From an application-focused perspective, follow-up studies should also focus on long-term perfusion, disease-specific stimulation, chronic exposure to inflammatory or toxic compounds, and validation of drug responses.

To the best of our knowledge, there are no studies that combine the use of fabrication technologies and living microfluidics to yield an in vitro model proposal that contains a macroporous trabecular bone, connected to a synovial capsule containing articular cartilage and synovial lining through an osteochondral interface, as described in this study. The proposed model is set apart by using more complex fabrication strategies to improve cell differentiation and increase fluid flow to the cells in the platform. Furthermore, it is fabricated with PDMS, a material widely used in the fabrication and mechanical actuation of microfluidic platforms. Combining our developed model with the use of complex biofabrication technologies as VBP, advanced cell sourcing for personalized medicine, the feasibility of culturing and differentiating the osteochondral tissues in the bioreactor platform, and potential advances in mechanical actuation, our models hold promise to advance OA research.

## Conclusions

We have successfully presented a proof-of-concept of a dually perfused bioreactor platform that contains 4 tissues that are critical to the architectural organization of the native knee joint and that can potentially be used to study the early-onset of OA. By integrating a volumetrically bioprinted macroporous trabecular bone, an osteochondral interface, articular cartilage derived from ACPCs, and a synovial lining within a single modular platform, this work provides a foundation for the development of more physiologically relevant joint models. The use of independently perfusable compartments further enables controlled fluidic access to distinct tissue regions. This complex and highly relevant model holds promise for the integration of several tissue compartments in a single microfluidic setup to further recapitulate the native synovial joint.

## Supplementary Material

Supplementary material associated with this article can be found in the online version at doi:10.1016/j.jcjp.2026.100328.

SUPPLEMENTARY INFORMATION
