# Supplementary material for: Engineering a dually perfused miniaturized bioreactor platform to enable multi-tissue knee joint modeling in vitro
Source: J Cartil Jt Preserv. Author manuscript; Available in PMC 2026 Nov 13. (PMC7619701; doi:10.1016/j.jcjp.2026.100328)
Supplement: Supplementary file 1 [file EMS221916-supplement-SUPPLEMENTARY_INFORMATION.pdf]

SUPPLEMENTARY INFORMATION

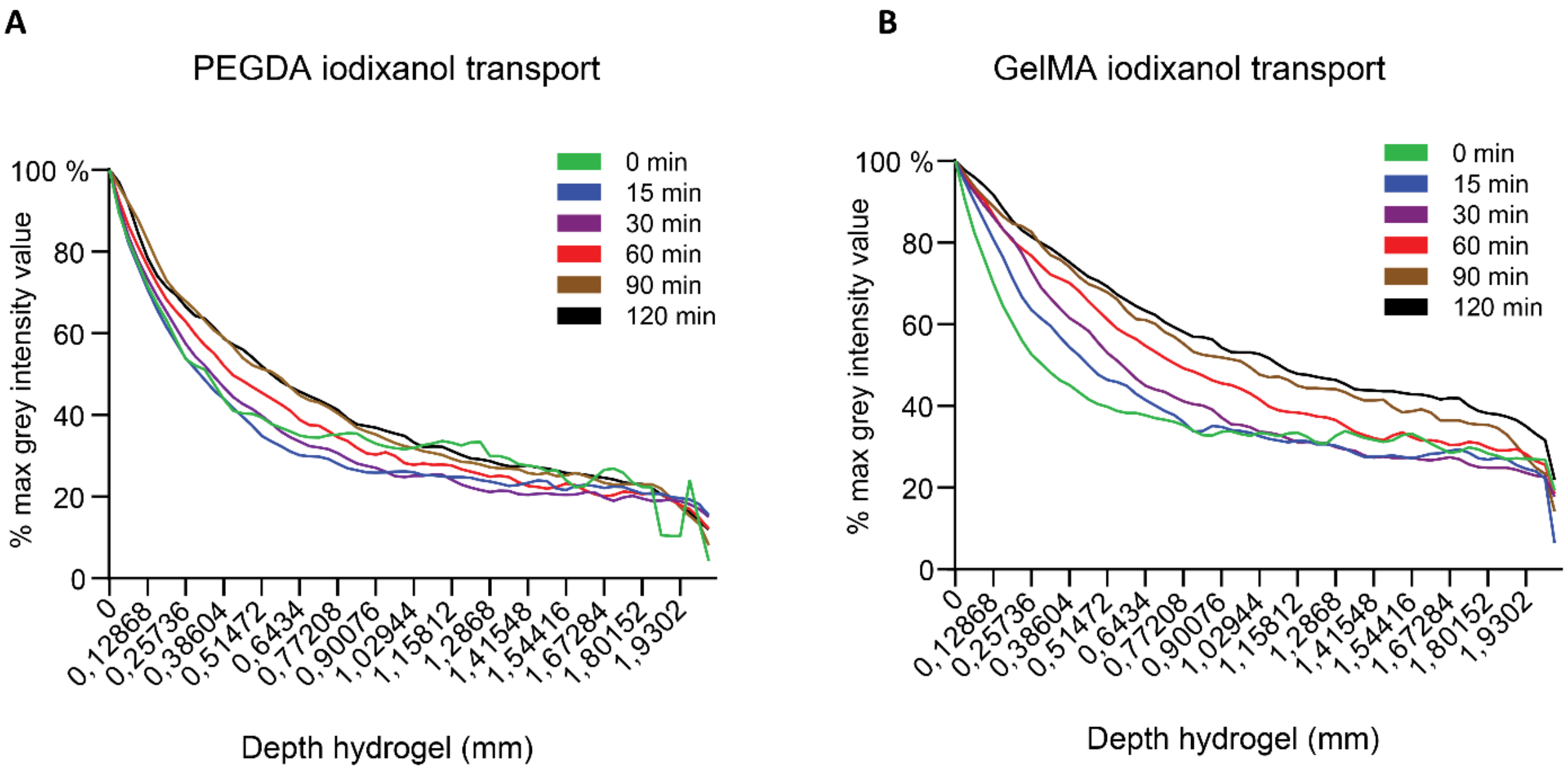

Supplementary Figure 1.- % maximum grey intensity value at midpoint of hydrogel at diverse timepoints. MicroCT scans of iodixanol infusion through hydrogel, n = 4.

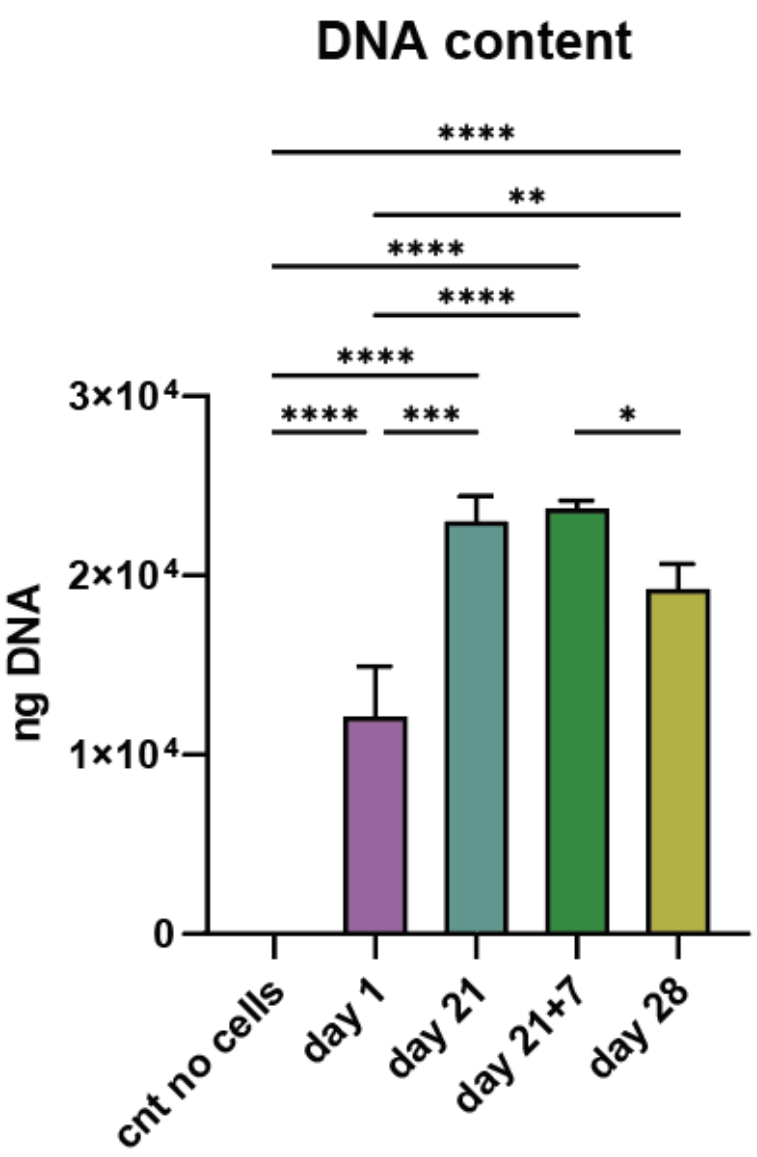

Supplementary figure 2.- Double-stranded DNA quantification n = 3.
